# Draft Genome Sequences of 63 *Pseudomonas aeruginosa* Isolates Recovered from Cystic Fibrosis Sputum

**DOI:** 10.1128/genomeA.00231-16

**Published:** 2016-04-21

**Authors:** Theodore Spilker, John J. LiPuma

**Affiliations:** Department of Pediatrics, University of Michigan Medical School, Ann Arbor, Michigan, USA

## Abstract

Here, we report the draft genome sequences of 63 *Pseudomonas aeruginosa* isolates, recovered in culture of sputum from 15 individuals with cystic fibrosis (CF) receiving care in a single CF care center over a 13-year period. These sequences add value to studies of within-host evolution of bacterial pathogens during chronic infection.

## GENOME ANNOUNCEMENT

Individuals with cystic fibrosis (CF) are susceptible to infection of the respiratory tract with *Pseudomonas aeruginosa*, which undergoes well-characterized phenotypic adaptation during chronic infection of CF airways (1). Recent studies have also noted considerable genetic variation among isolates serially recovered from CF patients during the course of chronic infection (2–4). This genetic variation may confound genotyping of *P. aeruginosa* isolates, which is important for monitoring potential interpatient spread of specific strains in this patient population. We determined the whole-genome sequences of 63 *P. aeruginosa* isolates cultured from 15 adult CF patients during a period of 13 years. The patients ranged in age from 19 years to 55 years (mean, 33 years). Between two and seven isolates were available from each patient; isolates were cultured from each patient during periods of time ranging from 4 to 13 years (mean, 6 years).

Bacteria were grown in Mueller-Hinton broth overnight at 37°C in an orbital shaker. Five ml of bacterial culture was pelleted, resuspended in 1 ml of 1X Tris-EDTA buffer and adjusted to an optical density of 0.55, corresponding to approximately 10^8^ CFU. Genomic DNA was extracted from 350 µl of the adjusted suspension using the MagNA Pure Compact nucleic acid isolation kit (Roche) following the manufacturer’s instructions. Genomic DNA libraries were prepared using an Illumina TruSEQ DNA library kit and sequenced on an Illumina HiSEQ 2500 paired-end flow cell (2 × 125-bp read length, V4 chemistry) at the University of Michigan Medical School DNA Sequencing Core. Output files containing the fastq reads were checked and edited using Trimmomatic version 0.33 (5). Read correction and assembly of draft genomes was carried out using SPAdes version 3.5.0 (6). Genomic alignments, phylogenetics, and single nucleotide polymorphism (SNP) visualization was performed using Gingr and Parsnp programs of the Harvest tools version 1.2 suite (7). SNP analysis was carried out using the ISG pipeline (8), employing MUMMER version 3.23 (9), BWA version 0.7.12 (10), and GATK version 3.4 (11). Genomes were annotated using NCBI’s whole-genome shotgun submission portal containing the automated Prokaryotic Genomic Annotation Pipeline (PGAP) option.

Draft genomes ranged in size from 6,118,548 bp to 6,884,695 bp, and contained 5,617 to 6,333 coding sequences (Table 1). The *N*_50_ of the draft genomes ranged from 111,223 to 588,702 bp, with 33 to 124 contiguous pieces. Pairwise comparison of draft genomes of isolates from any one patient revealed that SNPs ranged from a low of 43 and a high of 3,160 with a mean range of 771 and a median range of 719.

**TABLE 1:** Global statistics of draft genome sequences of 63 *Pseudomonas aeruginosa* isolates

Patient	Isolate^a^	Yr isolated	BioSample no.	Accession no.	Genome size (bp)	CDSs^b^	No. of contigs	*N*_50_
1	AU6462	2003	SAMN04436460	LRYR00000000	6,678,504	6,119	81	178,306
1	AU9381	2005	SAMN04436461	LRYD00000000	6,712,367	6,193	84	160,605
1	AU10014	2005	SAMN04436462	LRYE00000000	6,515,569	5,989	76	168,780
1	AU10015	2005	SAMN04436463	LRYF00000000	6,697,715	6,192	105	130,232
1	AU10714	2006	SAMN04436464	LRYG00000000	6,518,682	6,031	89	138,224
1	AU11866	2006	SAMN04436465	LRYH00000000	6,441,026	5,909	87	158,236
2	AU2342	2000	SAMN04436466	LRYI00000000	6,644,537	6,137	59	247,442
2	AU5471	2003	SAMN04436467	LRYJ00000000	6,576,117	6,061	48	417,292
2	AU10241	2005	SAMN04436468	LRYK00000000	6,517,933	6,000	57	333,007
3	AU6854	2004	SAMN04436469	LRYL00000000	6,746,537	6,202	66	276,987
3	AU9739	2005	SAMN04436470	LRYM00000000	6,861,787	6,327	66	195,665
3	AU20916	2010	SAMN04436471	LRYN00000000	6,837,226	6,300	70	231,549
4	AU6923	2004	SAMN04436472	LRYO00000000	6,490,208	6,056	57	233,457
4	AU12175	2006	SAMN04436473	LRYP00000000	6,529,487	6,105	76	193,222
4	AU13212	2007	SAMN04436474	LRYQ00000000	6,519,696	6,061	38	349,744
4	AU13213	2007	SAMN04436475	LRYS00000000	6,508,051	6,056	44	329,608
4	AU16960	2008	SAMN04436476	LRYT00000000	6,499,229	6,051	33	588,702
5	AU7032	2004	SAMN04436477	LRYU00000000	6,539,801	6,026	33	492,301
5	AU15431	2008	SAMN04436478	LRYV00000000	6,545,379	6,063	39	426,848
6	AU7033	2004	SAMN04436479	LRYW00000000	6,366,246	5,851	95	149,269
6	AU10583	2006	SAMN04436480	LRYX00000000	6,353,322	5,816	55	272,732
6	AU18068	2009	SAMN04436481	LRYY00000000	6,355,037	5,813	60	254,892
6	AU24156	2012	SAMN04436482	LRYZ00000000	6,356,852	5,808	59	232,432
7	AU7198	2004	SAMN04436483	LRZA00000000	6,548,147	6,081	91	130,397
7	AU10272	2005	SAMN04436484	LRZB00000000	6,588,605	6,093	53	202,738
7	AU10409	2005	SAMN04436485	LRZC00000000	6,475,849	5,985	56	184,471
7	AU10410	2005	SAMN04436486	LRZD00000000	6,580,210	6,096	56	202,738
7	AU10836	2006	SAMN04436487	LRZE00000000	6,536,502	6,110	124	111,223
7	AU16821	2008	SAMN04436488	LRZF00000000	6,572,055	6,095	54	253,358
8	AU8251	2004	SAMN04436489	LRZG00000000	6,293,365	5,797	56	209,586
8	AU12528	2006	SAMN04436490	LRZH00000000	6,327,593	5,810	49	233,151
8	AU17907	2009	SAMN04436491	LRZI00000000	6,336,865	5,820	54	211,550
9	AU9017	2005	SAMN04436492	LRZJ00000000	6,328,267	5,757	39	370,552
9	AU10502	2006	SAMN04436493	LRZK00000000	6,321,192	5,754	49	370,708
9	AU11990	2006	SAMN04436494	LRZL00000000	6,321,329	5,752	39	428,683
9	AU11991	2006	SAMN04436495	LRZM00000000	6,321,358	5,758	47	345,115
9	AU12424	2006	SAMN04436496	LRZN00000000	6,320,793	5,749	38	424,798
9	AU18274	2009	SAMN04436497	LRZO00000000	6,319,222	5,764	61	226,542
9	AU25116	2012	SAMN04436498	LRZP00000000	6,317,546	5,772	80	215,654
10	AU9899	2005	SAMN04436499	LRZQ00000000	6,240,563	5,706	64	213,685
10	AU13210	2007	SAMN04436500	LRZR00000000	6,239,781	5,688	49	318,484
10	AU19803	2010	SAMN04436501	LRZS00000000	6,158,966	5,617	52	232,854
10	AU19804	2010	SAMN04436502	LRZT00000000	6,161,159	5,626	66	179,368
11	AU10658	2006	SAMN04436503	LRZU00000000	6,341,036	5,836	77	176,289
11	AU17091	2008	SAMN04436504	LRZV00000000	6,264,463	5,733	35	355,061
11	AU21076	2010	SAMN04436505	LRZW00000000	6,230,429	5,700	64	193,145
11	AU25210	2012	SAMN04436506	LRZX00000000	6,268,083	5,733	38	335,848
12	AU10713	2006	SAMN04436507	LRZY00000000	6,262,596	5,719	51	238,345
12	AU17550	2009	SAMN04436508	LRZZ00000000	6,255,564	5,701	35	418,726
12	AU18132	2009	SAMN04436509	LSAA00000000	6,260,665	5,740	89	150,226
12	AU19319	2009	SAMN04436510	LSAB00000000	6,257,108	5,707	43	268,203
12	AU24526	2012	SAMN04436511	LSAC00000000	6,253,538	5,706	46	220,311
13	AU10756	2006	SAMN04436512	LSAD00000000	6,825,418	6,267	53	373,936
13	AU20671	2010	SAMN04436513	LSAE00000000	6,884,695	6,333	70	186,989
13	AU24807	2012	SAMN04436514	LSAF00000000	6,740,949	6,211	89	139,380
14	AU14820	2007	SAMN04436515	LSAG00000000	6,667,350	6,123	46	291,575
14	AU17965	2009	SAMN04436516	LSAH00000000	6,692,467	6,139	40	375,961
14	AU23529	2011	SAMN04436517	LSAI00000000	6,692,578	6,147	51	322,358
15	AU1215	1999	SAMN04436518	LRSF00000000	6,487,674	5,986	80	160,063
15	AU7511	2004	SAMN04436519	LRSG00000000	6,430,803	5,932	60	215,080
15	AU13626	2007	SAMN04436520	LRSH00000000	6,423,934	5,912	48	260,857
15	AU22632	2011	SAMN04436521	LRSI00000000	6,118,548	5,711	103	117,443
15	AU24787	2012	SAMN04436522	LRSJ00000000	6,150,557	5,707	61	192,465

^a^ All isolates are included in Bioproject PRJNA309533 and SRA SRP068878.

^b^ CDSs, coding sequences.

### Nucleotide sequence accession numbers.

This whole-genome shotgun project has been deposited in GenBank under the accession numbers listed in Table 1. The versions described in this paper are the first versions.
